# Norwegican Cartilage Project - a study protocol for a double-blinded randomized controlled trial comparing arthroscopic microfracture with arthroscopic debridement in focal cartilage defects in the knee

**DOI:** 10.1186/s12891-016-1156-y

**Published:** 2016-07-16

**Authors:** Tommy Frøseth Aae, Per-Henrik Randsborg, Anne Berg Breen, Håvard Visnes, Søren Vindfeld, Einar Andreas Sivertsen, Sverre Løken, Jan Brinchmann, Heidi Andreassen Hanvold, Asbjørn Årøen

**Affiliations:** Department of Orthopedic Surgery, Kristiansund Hospital, Kristiansund, Norway; Department of Orthopedic Surgery, Akershus University Hospital, Lørenskog, Norway; Department of Orthopedic Surgery, Ålesund Hospital, Ålesund, Norway; Department of Orthopedic Surgery, Haukeland University Hospital, Bergen, Norway; Department of Orthopedic Surgery, Haraldsplass Deaconess Hospital, Deaconess, Norway; Department of Orthopedic Surgery, Diakonhjemmet Hospital, Diakonveien, Norway; Department of Orthopedics, Oslo University Hospital, Oslo, Norway; Department of Immunology and Norwegian Center for Stem Cell Research, Oslo University Hospital, Rikshospitalet, Oslo, Norway; Department of Physiotherapy, Akershus University Hospital, Lørenskog, Norway; Institute of Clinical Medicine, University of Oslo, Oslo, Norway; Oslo Sports Trauma Research Centre, Norwegian School of Sport Sciences, Oslo, Norway

## Abstract

**Background:**

Focal lesions to the articular cartilage in the knee might have demolishing consequences to the knee. There exists a wide range of possible surgical procedures targeting these injuries, however no significant differences have been found between these procedures. This may support that the improvement is a result of rehabilitation, and not the surgery itself. Arthroscopic microfracture (MF) treatment has gained popularity, and has become the treatment of choice in patients with knee cartilage defects globally. In this study we want to increase knowledge, both clinical and economic, about arthroscopic microfracture (AF) compared to arthroscopic debridement (AD) and physical rehabilitation both in the short run, and in the long run.

**Methods/Design:**

To compare arthroscopic microfracture with arthroscopic debridement and physiotherapy for the treatment of focal cartilage lesions in the knee, a long-term, double-blinded, randomized controlled multicenter trial will be conducted. A total of 114 men and non-pregnant women with a symptomatic focal full thickness cartilage lesion in the knee less than 2 cm2 will be included in the study. The two treatment allocations will receive identical rehabilitation, which is made up of 3 phases: accommodation, rehabilitation and return to activity. Follow up is 24 months, where all will be invited to participate in late follow ups after 5 and 10 years.

The Knee Injury and Osteoarthritis Outcome Score (KOOS) knee-related quality of life (QoL) subscore is the primary endpoint. Clinical parameters, questionnaires and radiologic modalities (Magnetic Resonance Imaging (MRI) and x-ray) will be used as secondary endpoints.

**Discussion:**

This is an ongoing multicenter study with a high level of evidence to compare arthroscopic microfracture with arthroscopic debridement and physiotherapy for the treatment of isolated symptomatic full thickness cartilage lesions in the knee joint.

**Trial registration:**

ClinicalTrials.gov ID: NCT02637505 (December 15, 2015).

## Background

Articular cartilage is highly specialized tissue in joints, with main functions to provide lubrication to the joint and load shearing. Since the hyaline cartilage covering the articular surface is devoid of blood vessels, lymphatic drainage and nerves, very limited intrinsic healing capacity exist [1–3]. Focal lesions to the articular cartilage in the knee might have demolishing consequences to the knee both in the short term and in the long term, due to the predisposition of early onset osteoarthritis. With a prevalence of 12 % in young adults, focal cartilage lesions are common [4]. Musculoskeletal disorders (MSDs) are the most common occupational disease in the European Union. Workers in all sectors and occupations can be affected, and this may have a major impact both socially and not least financially [5–8]. With this in mind, treatment of symptomatic articular cartilage lesions is of great interest to those who prevent and treat MSDs, as well as the patient and the society [3, 9, 10].

All types of articular cartilage repair procedures aim to repair cartilage whilst keeping options open for alternative treatments in the long term. The most common surgical interventions are arthroscopic debridement, bone marrow techniques with or without augmentation with stem cells (including microfracture and autologous matrix-induced chondrogenesis), osteochondral transplantation (autografts and allografts), cell based repairs (autologous chondrocyte implantation (ACI) and autologous mesenchymal stem cell transplant [11–13].

Arthroscopic debridement (AD) focusses on removing loose articular flaps and fibrous tissue to the subchondral bone [12]. Arthroscopic microfracture (AM) includes an AD procedure followed by drilling holes (micofractures) through the subchondral bone using an awl. This generates a blood clot (called a super-clot) which becomes fibrocartilage over time and fills the articular cartilage defect [12]. Improved knee function have been reported in some studies [14], but they have been criticized for weakness in methodology [15].

None of the various options for focal cartilage injuries have been able to demonstrate a superior outcome when compared to each other in randomized controlled trials, suggesting that the strict post-operative rehabilitation common for all techniques are more important to the clinical improvement than the cartilage surgery itself [16, 17].

Due to its minimally invasive approach, technical simplicity and low costs, microfracture treatment has gained popularity over the past decades, and has become the treatment of choice in patients with knee cartilage defects [18–23]. Microfracture is most likely not effective for larger lesions [22, 24–26], and is currently recommended for lesions below 2–4 cm^2^ [27–30]. Meta-analysis and systematic reviews have required well-designed, long-term, multicenter studies to evaluate clinical outcomes of microfracture with the use of a “no treatment” group as a control group to increase knowledge, both clinical and economic, about arthroscopic microfracture compared to arthroscopic debridement in the short and long run [10, 25, 26, 31, 32].

### Purpose

Comparing arthroscopic microfracture (AM) with arthroscopic debridement (AD) in patients with symptomatic full thickness knee cartilage defects less than 2 cm^2^ in the knee joint is the purpose of this study. The aims will be differences both in regard of subjective and objective variables at predefined times up to 24 months follow up.

## Methods and design

The study emanates from “The Norwegian Cartilage Project” (NCP). The NCP is a Norwegian project organization with a goal of improving the treatment of injured articular cartilage in the knee through five studies. The study protocol is written in collaboration with the authors behind the already published study protocol regarding ACI and debridement [33].

**Fig. 1:**
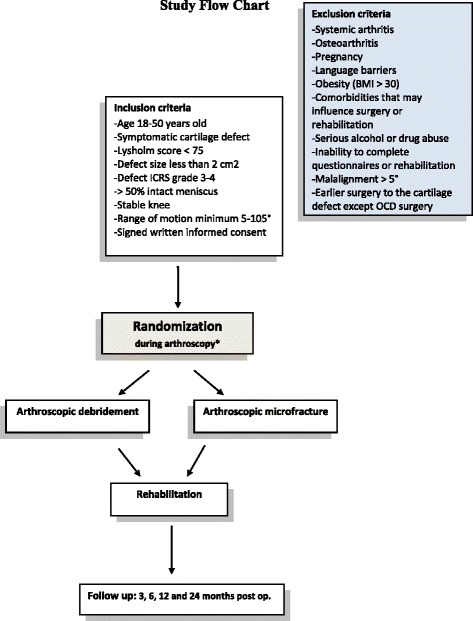
Study flow chart

This multicenter study with 2 treatment arms (AM and AD) is designed as a prospective, randomized, double-blinded parallel study, see Fig. 1. The study will be conducted at Norwegian hospitals including Kristiansund Hospital, Haukeland University Hospital, Ålesund Hospital, Diakonhjemmet Hospital, Haraldsplass Deaconess Hospital, Oslo University Hospital – Ullevål and Akershus University Hospital. Each hospital will have a member of the NCP group as a local coordinator. The sponsor of the study is Akershus University Hospital, with sponsor representative professor Asbjørn Årøen.

Inclusion, treatment and follow-up of the patients will be done at the orthopedic clinic at each of the above mentioned hospitals. To secure double blinding at the 24 months follow up, the control will be performed by an external reviewer connected to the NCP group. Follow up is 24 months, but all will be invited to controls at 5 and 10 years respectively.

### Participants

A total of 114 non-pregnant women and men with the age between 18-50 years old will be enrolled in the study, with a cartilage defect up to 2 cm^2^ in the femoral condyles or trochlea. By using a Lysholm score less than 75 [34], we only include symptomatic defects in the study. Included patients will have normal alignment (varus or valgus less than 5° measured clinically and if uncertainty, on hip-knee-ankle (HKA) angle images), normal range of motion (ROM) (minimum 5–105°), a stable knee with no sign of radiologically osteoarthritis [35] obtained with the SynaFlex X-ray positioning frame [36]. The inclusion criteria are based on Steadman et al, Saris et al and Ulstein et al [22, 37, 38]. Prior to inclusion, written informed consent will be obtained from all subjects.

All patients will, prior to inclucion, be assessed clinically and radiologically. Using the International Cartilage Repair Society (ICRS) grading system of cartilage defects, the defect will be verified arthroscopically as a grade 3 or 4 [39]. Peroperatively arthroscopic findings such as inappropriate size of the lesion, osteoarthritis or less than 50 % normal meniscus will lead to preoperatively exclusion [40]. The operating surgeon will perform definitive inclusion of the patient in the study. According to the standard at the local hospital, excluded patients will receive appropriate treatment.

Systemic arthritis, osteoarthritis, pregnancy, language barriers, severe obesity (body mass index > 30), co-morbidities that may influence surgery or rehabilitation, alcohol, substance or drug abuse, inability to complete questionnaires or rehabilitation and psychiatric disorders are exclusion criteria, see Table 1. Previous surgery to the cartilage defects except debridement and fixation of osteochondritis dissecans lesions are also exclusion criteria. Previous alignment procedures, cruciate ligament reconstruction and meniscus surgery are not criteria for exclusion. Those who withdraw during the trials or declining enrollment will be treated according to the standard of care.

**Table 1:** Inclusion and exclusion criteria

Inclusion criteria	Exclusion criteria
-Age 18–50 years old -Symptomatic cartilage defect -Lysholm score < 75 -Defect size less than 2 cm^2^ -Lesion graded ICRS 3–4 - > 50 % intact meniscus -Stable knee -Range of motion 5–105° -Signed written informed consent	- Systemic arthritis - Osteoarthritis - Pregnancy - Language barriers - Obesity (BMI > 30) - Comorbidities that may influence surgery or rehabilitation - Serious alcohol or drug abuse - Inability to complete questionnaires or rehabilitation - Malalignment > 5° - Earlier surgery to the chondral defect excluding OCD surgery

### Blinding and randomization

The study is designed as a double blinded study. As long as the trial lasts, the patients will not verbally nor written be informed which treatment is given to them. The operating surgeon will not be blinded, but will not be involved in the follow ups. Doctors involved in follow ups will be blinded for the given treatment. When patients are completing the questionnaires during follow ups, the case report forms (CRF) will not contain any information of the given treatment. The follow up at 24 months will be performed at each of the hospitals by an external reviewer connected to the NCP group to secure double blinding at 24 months follow up. The analysis of X-rays after 24 months will be done by an orthopaedic surgeon and a study radiologist, both blinded for the given treatment. The MRI at 24 months will be analyzed by the same blinded study radiologist.

By using a computer generator (randomization.com), the 114 participants will be randomized in blocks of six-eight (1:1) according to treatment allocations. All included participants will be given a number from 1 to 114 on inclusion. Concealed in opaque numerically marked envelopes, randomization will be printed in faded text. An employee at Kristiansund Hospital, not involved in the study, will administer the printing and concealing. The randomization will be done in the operation room during surgery, after the lesion has been graded and measured by the operating surgeon (see section “Operative procedure”).

### Arthroscopic procedure

No tourniquet is used. Three standard incisions are made. The procedure starts with a diagnostic arthroscopic examination to verify that the inclusion criteria are present. Any other necessary procedures (plica, meniscus, removal of loose bodies) are performed. Removal of lose and marginally attached cartilage is then done. Thereafter, the cartilage lesion is classified according to the ICRS classification, and measured using a standard 4-mm arthroscopic probe [39, 40]. The randomization is performed at the end of the arthroscopy (see section “Blinding and randomization) and the lesion will either undergo arthroscopic debridement (AD) or arthroscopic microfracture (AM).

#### Arthroscopic debridement

The lesion is stabilized, debriding all loose or marginally attached cartilage from the surrounding rim to form a stable edge of healthy cartilage around the defect using a ring curette, where cartilage slops down to the defect.

#### Arthroscopic microfracture

The cartilage is cut sharp forming a rim of 90°. The calcified layer is removed using a curette before an arthroscopic awl is used to perform multiple holes (“microfractures”) from the periphery towards the center. The microfractures are 3–4 mm apart and 2–4 mm deep into the subchondral bone. The correct and successful technique is confirmed by direct observation of marrow fat droplets exiting the microfracture as the fluid pump is reduced.

The instruments are removed and the incisions are sutured. Since local anesthetics may have a harmful effect on cartilage, intra-articular local anesthetics will not be used [41–43]. These surgical principles follow the techniques by Steadman and Mithoefer [24, 44–46]. Before study start, all surgeons will be given proper training in the arthroscopic study procedure.

### Post-operative protocol

Identical postoperative care will be given, usually with hospital admission up to 4 days. If there is a risk of thromboembolic disease, anti-thrombotic prophylaxis is given. Prophylactic antibiotic is not given. Up to 2 weeks of sick leave after the operation is given all participants. Any complications that should occur will be managed in a prompt manner by the involved departments.

### Rehabilitation protocol

All patients will be subjected to the same postoperative rehabilitation protocol, based on the work by Wondrasch et al [17]. The rehabilitation program will be headed by a designated study physiotherapist along with the patient’s local physiotherapist. The rehabilitation protocol involves three phases: accommodation, rehabilitation and return to activity, and is identical to the rehabilitation protocol in the previous mentioned ACI study [33], see Table 2.

**Table 2:** Rehabilitation protocol (identical for both groups)

Rehabilitation phases	Objectives	Physiotherapy and activities	Criteria for progression to next phase
1^st^ phase: Accomodation	- Reduce symptoms^a^ - Normalize ROM og muscle control	- Education/coaching - ROM, isometric exercises - Gait training (no weight-bearing for two weeks)	- No symptoms during ADL - Flexion 90° - Normalized quadriceps
2^nd^ phase: Rehabilitation	- Full ROM - Normalize muscle strength and joint stability	- Stationary bike cycling - Progressive knee and hip resistance training - Neuromuscular training	- Full ROM - No symptoms after training - Equally distributed weight on the lower limbs during weight-bearing exercises - Ability to stand on 1 limb on a flat surface for at least 10 s
3^rd^ phase: Return to activity	- Recovery of strength and neuromuscular control - Return to activity/sport	- Knee and hip resistance training - Neuromuscular training - Cardiovascular training	- Return to activity/sport based on individual assessment

^a^ Symptoms = pain and swelling

A designated hospital physiotherapist and the surgeon will on the first postoperative day instruct the patients in range of motion exercises and gait training according to Phase 1. Within two weeks of surgery, the patient is seen by a local physiotherapist, who will supervise the rehabilitation program in liaison with the study physiotherapist.

The rehabilitation program includes both active rehabilitation and patient education, and consists primarily of knee/hip progressive resistance, neuromuscular training and cardiovascular resistance, including plyometric exercises and balance. During the physiotherapy, the patients will be explained how to perform the exercises and why they are important, adjusted to pain or other symptoms.

All patients are asked to use training diaries to provide information about the training habits. Additionally, every second week patients are asked to respond to an online survey consisting of 4 questions, all with both predefined answers (closed answers) and open answers available. The training dairy will be continued till the end of the project (24 months), while the online survey continues as long as the patients are under supervision of a physiotherapist.

- 1^st^ phase - Accomodation:
  During admission in hospital, the leg is placed in a continunous passive motion (CPM) machine as tolerated, aiming to achieve 30–70° on the first postoperative day. The CPM should be used up to 6–8 h every 24 h during the hospital stay.
  For the first two weeks, only touch-down weight-bearing using crutches is allowed. The patients are encouraged to continue range of motion exercises, existing of 500 knee extensions/flexions three times a day, after discharge from hospital. Each patient should be scheduled to 2 to 3 supervised physical therapy sessions. When the wound is healed, swimming is allowed.
  Passing two weeks, weight-bearing as tolerated is carefully introduced, and gradually increased up to full weight-bearing. When the patient walks normal with limping, crutches are not needed.
- 2^nd^ phase - Rehabilitation:
  The patients perform 1–2 unsupervised training sessions per week, and attend at least 2 supervised physical therapy sessions. Exercises are performed with the uninjured and injured limb. Walking with increasing distances is encouraged, and when full weight-bearing is achieved, cross-country skiing can be allowed.
- 3^rd^ phase – Return to activity/sport:
  The patients perform unsupervised resistance training for a minimum of 2 and a maximum of 4 sessions per week, and attend at least 1 supervised physical therapy sessions per week. According to the goals for each patient, this phase is individualized. If return to sport is planned, sport-specific activities are included as functional progressions in the rehabilitation protocol.

### Outcome measures

Outcome measures will be the same as the endpoints in an earlier mentioned RCT coming from the same NCP group [33], enabling a direct comparison in outcome.

### Demographics

Age, gender, nationality, social status, work status, use of medication, prior medical history height in cm, weight in kg, Body Mass Index (BMI) and injury mechanism (if any) are collected at inclusion.

### Endpoints

The Knee Injury and Osteoarthritis Outcome Score (KOOS) knee-related quality of life (QoL) subscore is the primary endpoint. Validated to use in cartilage research studies, KOOS is a patient reported outcome measure [47], and will enable comparison of our results with other knee cartilage studies. At 24 months follow up, the difference in KOOS QoL subscore in the AM group compared to the AD group is the primary aim. No interim analysis is planned.

Clinical parameters, questionnaires and radiologic modalities will be used as secondary endpoints at predefined times at 3 months (±2 weeks), 6 months (±4 weeks), 12 months (±6 weeks) and 24 months (±8 weeks). They include range of motion (ROM) measured with a goniometer, visual analogue scale (VAS), all KOOS subscores except knee-related QoL, Tegner score, Lysholm score and EQ-5D. The difference between the two treatment groups and within the group will be the secondary aims. Information about work (return to work), physical activity and return to sport will also be collected.

Additionally, at the follow up after 24 months, a hop test validated by Reid [48], standing radiographs and MRI will be done. All participants will be invited to late follow ups after 5 and 10 years respectively.

### Hypothesis

Null hypothesis: There is no difference in KOOS QoL following AM or AD treatment of a symptomatic full thickness knee cartilage defect less than 2 cm^2^ 24 months after surgery.

Alternative hypothesis: There is a difference in KOOS QoL following AM or AD treatment of a symptomatic full thickness knee cartilage defect less than 2 cm^2^ 24 months after surgery.

### Statistical analysis

Demographic variables will be presented as mean ± standard deviation (SD) for continuous variables, and frequencies for categorical variables. The normal distribution of the variables will be tested. Given normal distribution, the statistical analysis will be performed using linear mixed models (LMM), including a random center effect for hospital. The test for the primary objective of comparing KOOS QoL from baseline to 24 months between the groups can be performed as a post hoc *t*-test following the LMM. If the data is not normally distributed, a non-parametric analysis will be done using the Mann-Whitney *U*-test. To evaluate the efficacy of treatment in terms of categorical outcome measures we plan to use Chi-squared tests. To adjust for possible confounders such as age of the patient and severity of the cartilage lesion, these variables will be included in the LMM. A *p* < 0,05 is considered statistically significant. The statistical analysis will be conducted in collaboration with a statistician at Unit for Applied Clinical Research at Norwegian University of Science and Technology, using SPSS v.22 (SPSS Inc, Chicago, Illinois) and R [49].

### Sample size

A difference in KOOS QoL subscore of 8–10 has previously been shown to be clinically significant [50, 51]. With 80% power comparing the two treatment groups 24 months after surgery by a two-sample *t*-test, power analyses is therefore based on detecting a difference of 10. Using a standard deviation (SD) of 18 ([37, 50] http://www.koos.nu/koosfaq.html), this yields 52 patients in each group. By adding 10 % due to drop out and loss to follow-up, a total of 114 patients will be included.

### Risk assessments

Participants may find it discomforting and unpleasant when they are asked about demographic information. The treatment of choice in this patient category is an arthroscopic operation, so no additional risks exists in participating this study. With a frequency < 1 %, these risks is rare and include arthralgia, joint effusion/swelling deep vein thrombosis (DVT), infection, headache and nasopharyngitis. Both treatments (AD and AM) have a risk of failure, and some might be in need of further surgery.

If any complication occurs during surgery, postoperative management and rehabilitation, patients will be examined by the doctor on call and treated according to clinical guidelines.

## Discussion

The treatment of isolated articular cartilage lesions of the knee is an orthopaedic puzzle, with numerous surgical techniques. Arthroscopic microfracture treatment has become the treatment of choice in patients with knee cartilage defects, but current knowledge is hampered by the lack of well-designed randomized studies comparing the effect of microfracture with debridement alone. This study will answer some of the questions regarding the benefit of AM compared to physical rehabilitation alone, and the results may help surgeons improve clinical outcome after articular cartilage injuries of the knee.

## Abbreviations

ACI, autologous chondrocyte implantation; AD, arthroscopic debridement; ADL, activity of daily living; AHUS, Akershus University Hospital; AM, arthroscopic microfracture; BMI, body mass index; CI, confidence interval; CPM, continuous passive motion; CRF, case report form; DVT, deep vein thrombosis; EQ5D, european quality of life five dimensions; HKA, hip knee ankle; ICRS, International Cartilage Repair Society; ISF, investigator site file; ISPOR, International Society for Pharmacoeconomics and Outcomes Research; KOOS, knee injury and osteoarthritis outcome score; LMM, linear mixed models; MRI, magnetic resonance imaging; MSDs, musculoskeletal disorders; NCP, Norwegian Cartilage Project; OCD, osteochondritis dissicans; PROMS, patient reported outcome measures scores; QoL, quality of life; RCT-CEA, randomized clinical trial-cost-effectiveness analysis; ROM, range of motion; SD, standard deviation; SPSS, Statistical Package for the Social Sciences; VAS, visual analogue scale
